# Impact of Silver Nanoparticle Treatment and Chitosan on Packaging Paper’s Barrier Effectiveness

**DOI:** 10.3390/polym16152127

**Published:** 2024-07-26

**Authors:** Dimitrina Todorova, Nikolay Yavorov, Urška Vrabič-Brodnjak

**Affiliations:** 1 Department of Pulp, Paper and Printing Arts, Faculty of Chemical Technology, University of Chemical Technology and Metallurgy, 1797 Sofia, Bulgaria; todorova.dimitrina@uctm.edu (D.T.); nyavorof@uctm.edu (N.Y.); 2 Department of Textiles, Graphic Arts and Design, Faculty of Natural Sciences and Engineering, University of Ljubljana, 1000 Ljubljana, Slovenia

**Keywords:** silver nanoparticles, chitosan, coating, packaging paper, antibacterial

## Abstract

In this study, a comparative analysis of silver nanoparticles treatment and chitosan coating on packaging paper barrier properties was carried out. In order to examine the water, grease, and antibacterial barrier properties of silver nanoparticle-treated and chitosan-coated laboratory-obtained paper samples, a mixture of bleached softwood and hardwood celluloses was used. In order to conduct the comparative analysis SEM, water contact angle, Cobb_60_, and Kit tests were carried out on a cellulose sample, and four paper samples (three of them treated with silver nanoparticles—1, 2, and 3 mL/20 cm^2^ or chitosan coated—0.5, 1, and 2 g/m^2^) together with the inhibition activity against nine Gram-positive and Gram-negative bacteria, yeast, and fungal strains. The study found out that increasing the silver nanoparticle treatment and chitosan coating led to improved water resistance, while grease resistance was improved only for chitosan coated paper samples. Additionally, paper treated with 3 mL/20 cm^2^ of silver nanoparticles had the highest antibacterial protection (81.6%) against the Gram-positive bacterium *Staphylococcus aureus*, followed by Gram-negative *Escherichia coli* (75.8%). For the rest of the studied microorganisms, the average efficiency of the treated paper was 40.79%. The treatment of the paper with 1 and 2 mL/20 cm^2^ of silver nanoparticles was less effective—27.13 and 39.83%, respectively. The antibacterial protection of 2 g/m^2^ chitosan-coated paper samples was the most effective (average 79%) against the tested bacterial, yeast, and fungal strains. At 1 and 0.5 g/m^2^ chitosan coatings, the efficiency was 72.38% and 54.67%, respectively. Gram-positive bacteria, yeasts, and fungal strains were more sensitive to chitosan supplementation.

## 1. Introduction

The search for sustainable and effective packaging solutions has attracted considerable attention in recent years, particularly in the food industry where packaging materials must balance environmental impact with functional performance [1,2]. Conventional petroleum-based packaging materials offer effective barrier properties but pose a significant environmental problem due to their non-biodegradability. This has spurred research into alternative materials that are both effective and environmentally friendly. Two promising avenues in this regard are the use of silver nanoparticles (AgNPs) and biopolymer coatings, such as polysaccharides (chitosan), which are known for their antimicrobial properties and potential to improve the barrier function of packaging materials [3,4,5,6].

AgNPs have attracted great interest in the field of antimicrobial packaging due to their unique physicochemical properties, especially their high surface-to-volume ratio, which enhances their interaction with microbial cells [7,8,9]. The antimicrobial mechanism of AgNPs primarily involves the release of silver ions, which can penetrate microbial cell membranes, disrupt cell function, and ultimately lead to cell death. This strong antimicrobial activity makes AgNPs an effective component of food packaging materials where the prevention of microbial contamination is critical to extending the shelf life of perishable products [4,10]. AgNPs can be incorporated into packaging materials by various methods. These include embedding in polymers, the formation of composites or application as a surface coating. Ag–water treatments, in which AgNPs are suspended in water and then applied to packaging substrates, are particularly promising. These treatments not only provide a robust antimicrobial barrier but also maintain the overall barrier properties of the packaging material against moisture and gases based on the additives used, such as alkyl ketene dimer (AKD) or alkenyl succinic anhydride (ASA), which are mainly used for paper hydrophobicity obtaining through their suspension or surface application. When incorporated into packaging materials, AgNPs can inhibit the growth of bacteria, fungi, and other microorganisms on the surface of the material. This microbial inhibition can indirectly improve the barrier properties by preventing degradation of the packaging material caused by microbial activity, which could otherwise increase permeability to moisture and gases. The incorporation of AgNPs can lead to a more compact and dense material structure. The nanoparticles can fill the micro-voids within the polymer matrix, reducing the free volume through which gases and moisture can permeate. This densification can enhance the material’s barrier properties. Studies have shown that packaging materials treated with AgNPs have a significant reduction in microbial load, including bacteria such as *Escherichia coli* and *Staphylococcus aureus*, thereby improving food safety and shelf life [11,12,13,14].

Researchers have been exploring various methods to integrate AgNPs into packaging materials to ensure uniform distribution and sustained antimicrobial activity [12,13,15,16,17]. Techniques such as electrospinning, solvent molding, and layer-by-layer assembly are currently being evaluated. The compatibility of AgNPs with various polymers, including biodegradable polymers such as polylactic acid (PLA) and polyhydroxyalkanoates (PHA), is an important area of research for the development of environmentally friendly antimicrobial packaging solutions as presented by the researchers Liao et.al, [18], Mulla et al. [19], and Samrot et al. [20]. Moreover, the effect of AgNPs on the barrier properties of packaging materials were presented by He et al. [21]. Studies are being conducted to investigate how the addition of AgNPs affects the water vapour transmission rate (WVTR) and oxygen transmission rate (OTR) of packaging films [11,22,23,24]. The aim was to optimize the AgNP concentration and distribution in order to achieve the best combination of antimicrobial activity and barrier performance. Comparative studies between AgNP-treated packaging and other antimicrobial treatments, such as organic acids or essential oils, are essential to identify the most effective strategies. In addition, the synergistic effects of combining AgNPs with other antimicrobial agents and barrier enhancers will be investigated to create multifunctional packaging materials with superior properties. By better understanding the antimicrobial mechanisms of AgNPs and optimizing their integration into packaging materials, the research aims to develop innovative packaging solutions that not only improve food safety but also extend the shelf life of perishable products. 

Therefore, one of the promising antimicrobial solutions are polysaccharides such as chitosan. Chitosan is obtained from chitin, which is found in the exoskeletons of crustaceans, insects, and fungi. It is a versatile biopolymer known for its excellent biodegradability, biocompatibility and antimicrobial properties [25,26]. Chitosan’s unique properties arise from its cationic nature, which allows it to interact with negatively charged microbial cell membranes, leading to the destruction of the cell wall and the death of the microbes. This makes chitosan an effective antimicrobial agent that is particularly useful in food packaging to prevent contamination and spoilage. Chitosan’s ability to form a film allows it to be used as a coating on various substrates, including packaging paper [27,28,29]. When applied to packaging materials, chitosan increases mechanical strength, making the packaging more durable and resistant to mechanical, tensile stress [28,30,31,32,33]. Chitosan coatings also improve barrier properties by reducing the permeability to water vapor and gases, which is crucial for maintaining the freshness and quality of food. Studies have shown that chitosan-coated papers have significantly lower WVTR and OTR, thereby extending the shelf life of perishable goods [27,28,34,35]. It can also be modified or mixed with other materials to further improve its functional properties. For example, the addition of plasticizers such as glycerin can improve the flexibility and mechanical properties of chitosan films. Similarly, blending chitosan with other biopolymers or incorporating nanoparticles (e.g., silver or zinc oxide nanoparticles) can lead to composite coatings with better barrier and antimicrobial properties. These modifications are important for tailoring the properties of chitosan coatings to specific packaging requirements. The combination of chitosan with other materials to produce composite coatings is an important area of interest. As presented by Gürler et al. [36], Shan et al. [37], and Rohadi et al. [38], mixing chitosan with gelatin, starch, or alginate can improve the mechanical and barrier properties. The incorporation of nanoparticles such as AgNPs, titanium dioxide (TiO_2_) or clay nanoparticles is being investigated to improve antimicrobial efficacy and barrier performance [5,39,40,41]. These composite materials aim to utilize the synergistic effects of different components for superior functionality. Detailed studies are currently being conducted on the antimicrobial mechanisms of chitosan to better understand how it interacts with different types of microorganisms [39,40,42,43]. This research includes investigating the effects of molecular weight, the degree of deacetylation, and pH on the antimicrobial activity of chitosan. In addition, the efficacy of chitosan against a wider range of pathogens, including viruses and fungi, were investigated [42,43]. Researchers have also explored functional enhancements of chitosan coatings, such as the addition of natural extracts (e.g., essential oils and plant extracts) to increase antimicrobial activity and add antioxidant properties [44]. These improvements aim to create multifunctional packaging materials that not only protect against microbial contamination but also contribute to the overall quality and safety of the packaged food. 

By improving understanding of the properties of chitosan and optimizing its application in packaging materials, the development is focused on sustainable and effective solutions that meet the growing demand for environmentally friendly packaging in the food industry. Comparative studies between chitosan coatings and other antimicrobial treatments, such as coatings with silver nanoparticles, are essential to identify the most effective strategies to improve the barrier properties and safety of packaging materials. As part of the current research, the aim of our research was to optimize the application method and concentrations of both AgNPs and chitosan to achieve maximum efficacy. Different formulations and composite materials to improve the functional properties of the packaging were investigated. Comparative studies are crucial to find the most effective and sustainable solutions for packaging materials. This research aimed to conduct a comparative analysis of both AgNPs treatment and chitosan coating on the barrier properties of packaging paper. By systematically evaluating the effectiveness of these treatments, we aim to gain insights into their potential applications and limitations, thus contributing to the development of advanced, sustainable packaging technologies.

## 2. Materials and Methods

### 2.1. Materials and Preparation Procedures

Cellulose mixtures in ratio 80:20 for paper samples were prepared from two kraft bleached wood-free pulps. The softwood one was delivered by Svenska Cellulosa Aktiebolaget (SCA), Sweden, while the hardwood one was provided by Svilosa AD, Svishtov, Bulgaria. A separate cellulose refining process had been conducted using laboratory Valley beater (a laboratory Hollander-type beater), according to ISO 5264-1:1979 [45]. The obtained pulp mixture beating degree was 30 °SR (Schopper Riegler value), in accordance with ISO 5267-1:1999/Cor 1:2001 [46].

While obtaining base paper samples, two wet-end chemical additives were added sequentially: (1) alkylketendimer sizing agent—1% of o.d.f (Kemira^®^ Fennosize KD 157YC, Kemira Oyj, Helsinki, Finland) and (2) cationic retention additive—0.025% of o.d.f. (cationic-modified polyacrylamide with 11.106 g/mol molecular weight and +1.05 charge density delivered by Ciba Specialty Chemicals-Ciba^®^ Percol^®^ Co. (Basel, Switzerland)). 

For the laboratory papermaking simulation process, a Rapid–Köthen laboratory paper-sheet machine (Birkenau, Germany) was used. All samples were prepared according to ISO 5269-2:2004 [47], with base weight of 50 g/m^2^. The drying of the wet paper samples was at 95 °C under −90 kPa pressure with duration of 7 min.

The AgNPs treatment was applied to the obtained 20 cm^2^ base paper samples with 1, 2, and 3 mL consumption from a 20 cm distance. Papers were sprayed with the exact amount of ARCOL silver water with concentration of 10 mg/L = 0.01 g/L, delivered by Gal ET. ARCOL silver water is a solution of colloidal silver in purified water obtained through a process of electrolysis, in which silver ions are suspended in water. This colloidal silver is a solution of silver nanoparticles suspended in a liquid base (CSAgNPs). The sample can be considered on the nanoparticle scale, as the size of the dispersed phase particles is between 1 and 100 nm.

The chitosan for the base paper coating layer used, was Fluka (BioChemika, Tokyo, Japan) Crayfish Chitosan 28191 (2-Amino-2-deoxy-(1->4)-β-D-glucopyranan) with medium viscosity and acetic acid—99.8% with M = 60.05 g/mol. A quantity of 1 g of the chitosan was dissolved in 1% (*v*/*v*) aqueous acetic acid (100 mL), which was stirred and heated to 50 °C in the first 2 h on a magnetic stirrer at 300 rev/min for 24 h until completely dissolved. The obtained chitosan solution had a viscosity of 300 mPa∙s.

Paper sample coating was performed with the laboratory coating machine K Control Coater (RK PrintCoat, Royston, UK). The coating machine with automatic control used standard wire-wound bars to produce a uniform and repeatable coating. Stainless steel wet film deposit K-bar 120 µm with wire diameter 1.52 mm was used. The coating was done at ambient temperature. Coated samples were dried at t = 50 °C in a vacuum dryer with *p* = 0.08 MPa until its complete drying. The chitosan coated paper samples had coating layers of 0.5 g/m^2^, 1 g/m^2^, and 2 g/m^2^.

The experimental investigations were conducted with nine paper samples, as described in Table 1.

### 2.2. Methods

#### 2.2.1. Morphology Characterization of Cellulose Fibers and Paper Samples

The cellulose fiber morphology was characterized through a microscopic analysis after placing a small amount of pre-milled fibers in a porcelain pounder, drenching with 10 mL of 1% NaOH, and letting stand for 10 min. The following procedure involved rinsing on a fine metal mesh with distilled water. From the obtained fiber cellulose material, three evenly distributed visual fields were prepared on a glass slide. On each of the fiber fields, a drop of Herzberg’s reagent (Cl-Zn-I), according to ISO 9184-3:1990 [48], was placed, and again, the fibers were distributed very carefully. Then, the glass slides with the evenly distributed cellulose fibers were dried in a chamber at about 50 °C. After cooling, a thin glass slide was placed to cover the fiber samples free of air bubbles and accumulations. The stained fibers were observed using a VisiScope^®^ TL254T1 (VWR, Milano, Italy) microscope with an integrated digital camera at 100× magnification; objective: 10×/0.25 E-PLAN, eyepiece: WF10×/20 mm. The images were subsequently analyzed using Optika ProView software (version 2.0).

Preparing samples for SEM analysis included several steps. Firstly, paper samples were cut and glued with adhesive tape on the holder. A gentle air blower was used to clean the surface dust, which could be attached during the sample preparation. After that, the sputtering coating was performed. A thin layer of conductive material—gold—was applied using a sputter coater (Agar Scientific, Stansted, UK). This involved placing the sample in a vacuum chamber where an inert gas argon was ionized to dislodge atoms of the coating material, which were then deposited onto the sample. After that, the samples were prepared for the analysis with an SEM microscope. The SEM micrographs of the pulp, paper, and film surfaces were taken with a scanning electron microscope (JSM6060 LV, Jeol Ltd., Tokyo, Japan). The instrument operated at 10 kV and at magnifications of 500× and 1000×.

#### 2.2.2. Structural Properties

The grammage of all samples was determined in accordance with the ISO 536:2019 standard [49]; 10 samples of each paper were cut into a size of 10 × 10 cm and weighed. 

The thickness of the samples was measured with a digital micrometer (Mitutoyo Corporation, Kawasaki, Japan) to the nearest 0.001 µm at 10 random locations on each paper, as described in the standard method ISO 534:2011 [50]. 

Paper smoothness was determined by the Bekk method, according to standard ISO 5627:1995/Cor 1:2002 [51]. The smoothness of individual surfaces of paper was determined using a clamping pressure of approximately 100 kN/m^2^ and an anvil with an effective area of 10.00 ± 0.05 cm^2^.

Paper samples were analyzed at 23 °C under 50% relative humidity.

#### 2.2.3. Barrier Properties

##### Contact Angle and Wettability

The contact angle measurements on the examined paper samples were evaluated at room temperature (25 °C) using Theta Lite (Biolin Scientific AB, Gothenburg, Sweden). A droplet of deionized water was allowed to fall on the paper surface samples using a syringe Gastight (Hamilton Company, Reno, NV, USA) at a height of 10 mm over the previously leveled (using the optical system) surfaces, and a camera captured the process. Data were captured using the three-point sampling approach. Thus, for each sample, six values were obtained, which were subjected to statistical analysis according to the seven-step system. For the value of Student’s criterion, t = 5.959 was taken, corresponding to a probability of P = 0.1%. The measurements were accompanied by taking 12 photographs per second for 10 s. The wettability was calculated according to the ASTM D724-99:2003 standard [52]. For the wettability calculation (see Equation (1)), the water contact angle at 0.25 s and 10 s of contact were taken:

(1) $$W=\frac{A-a}{10},$$

where *W* is the wettability (°/s); *A* is the water contact angle at 0.25 s (°); *a* is the water contact angle at 10 s (°); and 10 is the time (s) for paper water absorption.

Water absorptiveness was determined with the Cobb_60_ value, as described in the standard method ISO 535:2023 [53], where a given amount (100 mL) of distilled water was in contact with 100 cm^2^ of the paper for 45 s. The weight differences were compared after and before water contact. A 10 kg roller was used while removing the excess of water between absorbent paper. For each paper, five sample tests were made. 

##### Grease Resistance Test (Kit Test)

The TAPPI T 559 cm-22 procedure was used for the grease resistance testing of the investigated paper samples, which is an updated and expanded variant of TAPPI UM 557 “Repellency of Paper and Board to Grease, Oil, and Waxes (Kit Test)”. A sequence of numbered reactants from 1 to 12 (varying in viscosity or “aggressiveness” and surface tension) was used. The most aggressive solution had the lowest viscosity, surface energy (22.0 mJ/m^2^), and contact angle but was the highest numbered and remained on the surface of the paper without causing failure. Solution No. 1 consisted of 100% castor oil. In solutions No. 2 to 10, the castor oil content was gradually reduced, while the solvent content of n-heptane and toluene increased; their ratio in the mixture was 1/1. Solution No. 11 contained only solvents in a ratio of 1/1, and solution No. 12 also only contained solvents with a different ratio of 45% toluene and 55% n-heptane. Starting with Kit solution No. 1, drops of the solutions fell onto the paper from a height of 4 cm, and after 15 s, they were removed with tissue paper or fabric. The Kit value was accepted when the highest-numbered solution corresponded to no footprint on the surface of the paper or board.

#### 2.2.4. Antimicrobial Testing

As test microorganisms were used as follows: two Gram-positive bacteria (*Staphylococcus aureus* ATCC 6538 and *Bacillus cereus* АТСС 10876), three Gram-negative bacteria (*Escherichia coli* ATCC 8739, *Pseudomonas aeruginosa* ATCC 9027, and *Salmonella abony* NTCC 6017), two yeast strains (*Candida albicans* ATCC 10231 and *Saccharomyces cerevisiae* ATCC 2601), and two fungal strains (*Aspergillus brasiliensis* ATCC 16404 and *Fusarium moniliforme*).

From each bacterial test microorganism, a 24 h culture was prepared. Vegetative material was taken using a wire loop and suspended in 10 mL of saline (0.9% sodium chloride solution (0.154 mol/L)). The cell concentrations of the suspensions prepared were about 10^3^ CFU/mL.

Analogously, the yeast and fungal suspensions were prepared, with a 48 h cultivation time for yeast suspensions and 120 h for fungal suspensions. With sterile tweezers in aseptic conditions, each paper sample of 5 × 5 cm was placed in a sterile petri dish. On each square, 0.1 mL of the prepared cell suspensions was dropped with sterile pipette and carefully spread over the surface of the paper samples; then, the square was placed in an incubation chamber for 2 h at 30–35 °C.

Aseptically with a sterile pipette, in every petri dish, 20 mL of soybean casein agar for bacteria or Sabouraud dextrose agar for yeast and fungi was dropped.

For bacteria growth, samples were cultured in an incubation chamber at 30–35 °C for 24–48 h and 20–25 °C for 48–72 h for yeast and 120 h for fungi. The colonies grown in the petri dishes were counted by a colony counter.

The effect of CSAgNPs treated and chitosan coated paper on the growth of test microorganisms was evaluated by the antimicrobial efficiency calculated by the Equation (2):

(2) $$\%Efficiency=\frac{N_{0}-N_{1}}{N_{0}}\times 100,$$

where *N*_0_ is the number of the colony-forming units in the control sample, while *N*_1_ is that in the sample paper treated with CSAgNPs coated with chitosan.

#### 2.2.5. Statistical Analysis

Statistical analysis was carried out using one-way ANOVA with a confidence level of 95% (*p* < 0.05) in Microsoft^®^ Excel 2016 using the data analysis ToolPak. All experiments were performed according to standards, and the results were expressed as the mean ± standard deviation.

## 3. Results and Discussion

### 3.1. Characterization of Cellulose Fibers and Paper Samples

In principle, the characterization of all packaging materials starts with their raw material characterization, as this is directly linked to the end-product properties. Subsequently, the suspension treatment, surface treatment, or coating could ensure a change in the properties, either flowingly or drastically. Aiming to obtain packaging material for household implementation, the desired paper properties are as follows: optimal surface properties, excellent barrier properties (hydrophobicity and/or grease resistance), as well as antibacterial inhibition efficiency, all well-balanced with the basic weight of the material.

**Figure 1:**
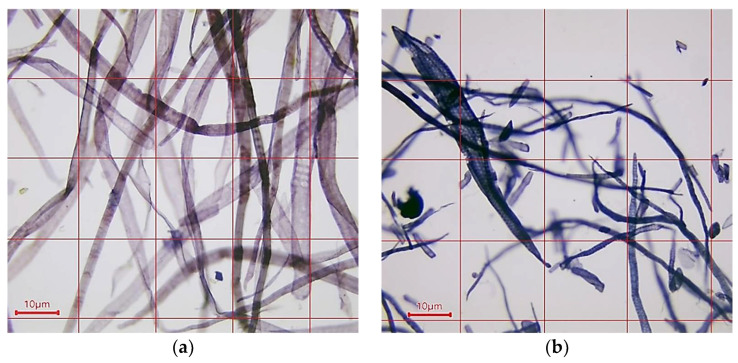
Microphotograph of a fiber raw materials taken at 100× magnification: (**a**) bleached kraft coniferous cellulose from pine tree; (**b**) bleached kraft deciduous cellulose from beech and poplar trees.

Thus, in this research of a fiber material, two types of virgin bleached kraft cellulose—soft and hardwood—were used. To characterize the exact wood type, a microscopic morphology characterization was carried out, and the results are illustrated in Figure 1. The softwood cellulose fibers (Figure 1a) that were analyzed were obtained from bleached kraft coniferous cellulose from pine trees, while the bleached kraft deciduous cellulose (Figure 1b) was obtained from beech and poplar trees.

**Figure 2:**
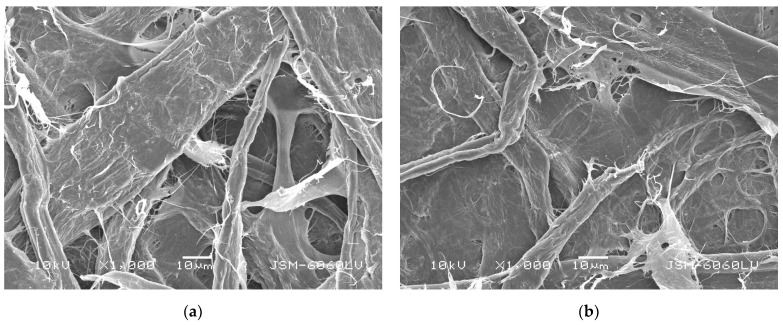
SEM micrographs taken at 1000× magnification and operating at 10 kV voltage: (**a**) OP; (**b**) BP (control).

The investigated cellulose fibers had a violet to bluish color (by the Herzberg’s reagent), indicating delignification to less than 6%. This result could indicate that strong hydrogen bonds connected the cellulose fibers in the resulting base paper samples, also conducted by the retention additive (modified cationic PAA) used, confirmed by the SEM images presented in Figure 2b. The resulting SEM images revealed smaller and less deep inter-fiber pores compared with the sheet material obtained by combining the soft- and hardwood fiber raw cellulose with no additives (see Figure 2a).

The paper sample characterization, especially for those obtained with surface treatment or coating, started with their grammage, thickness, and smoothness measurements, which are given in Table 2. All paper samples were laboratory-obtained on a sheet-casting apparatus with a basic weight in the range of 49–50 g/m^2^. The thickness of the paper samples also did not change significantly.

**Figure 3:**
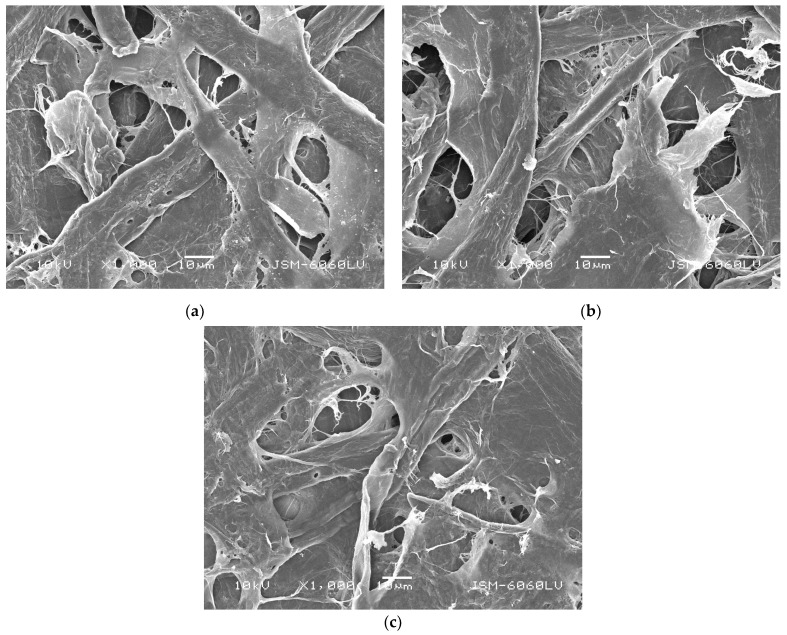
SEM micrographs taken at 1000× magnification and operating at 10 kV voltage: (**a**) BP/CSAgNPs 1; (**b**) BP/CSAgNPs 2; (**c**) BP/CSAgNPs 3.

Regarding the samples treated with CSAgNPs, an increase in the smoothness of the CSAgNPs treated papers was observed compared with the base paper, ceased by the additional moistening and its subsequent additional drying at 96 °C for 5 min, effecting as paper calendaring. As the amount of CSAgNPs increased, the effect decreased. Applying a water-based surface treatment caused to the paper material volume and porosity to increase, as cellulose is highly hygroscopic [54]. When a greater water-based treatment was performed, the smoothness decreased. This was also confirmed by the SEM images presented in Figure 3, as the paper pores were deeper, but the fibers were still interwoven with their typical structure, even at the maximum CSAgNP treatment consumption used (BP/CSAgNPs 3). This calendaring effect would probably occur in industrial production conditions too but would hardly be as visible as that under laboratory conditions.

**Figure 4:**
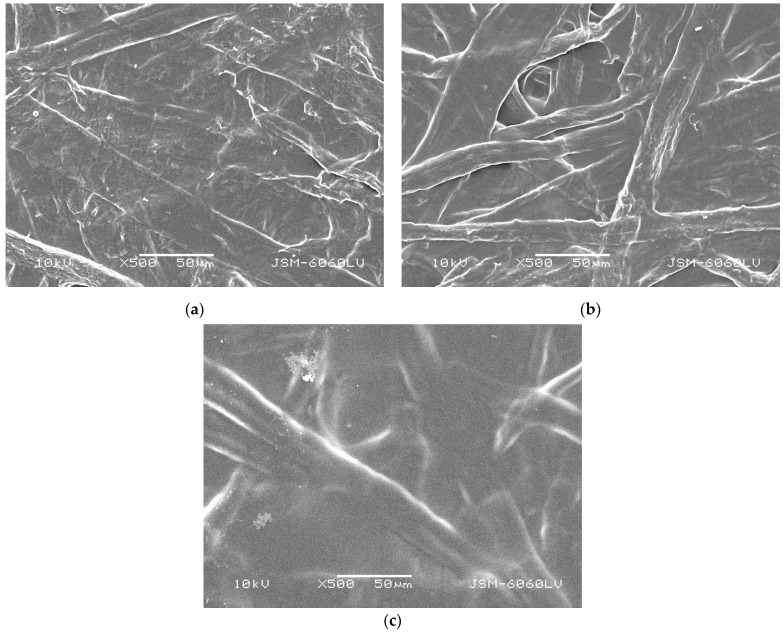
SEM micrographs taken at 500× magnification and operating at 10 kV voltage: (**a**) BP/Ch 0.5; (**b**) BP/Ch 1; (**c**) BP/Ch 2.

The paper coating process and its optimization depends on the smoothness of the base papers [55], as it is distinguished as point of reference for surface structure changes. The data in Table 2 reveals an increase in the chitosan-coated paper samples’ (samples BP/Ch 0.5, BP/Ch 1, and BP/Ch 2) smoothness. Taking both the Bekk analysis and the SEM images presented in Figure 2 and Figure 4 into account, we can conclude that the coating was successfully applied and shed, and the surface structure of all coated paper samples was dense, with an evenly distributed chitosan coating and closed paper pores. As the chitosan coating increased from 0.5 g/m^2^ to 2 g/m^2^ (samples BP/Ch 0.5, BP/Ch 1, and BP/Ch 2), the effect was enhanced.

### 3.2. Barrier Properties

According to many reports reviewed in [56], chitosan coatings have the ability to form films on the cellulose fiber surface, according to the structural similarity between chitosan and cellulose together with the specific chitosan properties, such as deacetylation degree, solution viscosity, coating concentration, and coating speed. Another fact that should be pointed out while choosing the coating layer grammage is the recommendation for a thinner coating layer [57], as to facilitate paper drying, both in the laboratory and industrially. On the basis of the above, the current experiment used chitosan with a medium viscosity (300 mPa∙s) and 99.8% acetic acid (M = 60.05 g/mol), applied in the smallest possible evenly distributed coating starting from 0.5 g/m^2^. Considering all the challenges and limitations, the preparation and comparative analysis between CSAgNP-treated and chitosan-coated papers with good barrier and antibacterial properties could contribute to the development of environmentally friendly packaging solutions.

The complex of barrier properties in relation to water, moisture, and grease is considered the main indicator of packaging papers. Undoubtedly, the most widespread hydrophobic/hydrophilic characteristic of papers, especially those for printing and packaging, is their surface adsorption capacity, expressed by the water contact angle, wettability, and Cobb_60_ water absorption index, g/m^2^, while for the grease resistance, the Kit test is widespread. 

**Figure 5:**
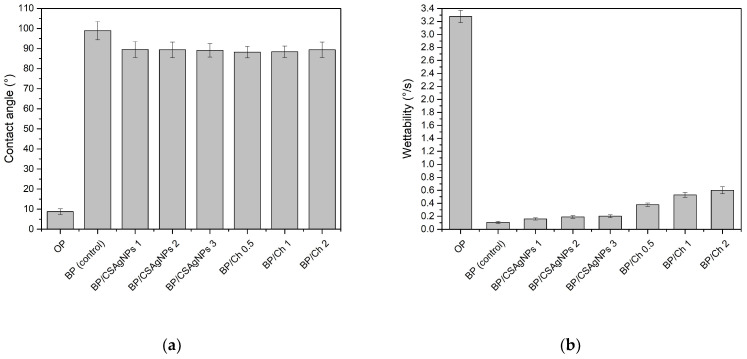
Contact angle (**a**) and wettability (**b**) of the cellulose, base papers, and CSAgNP-treated and chitosan-coated paper samples.

In order to compare the paper samples’ hydrophobicity of only the pulp mixture, base paper (control), and CSAgNP-treated and chitosan-coated paper samples, the contact angles and wettability of all eight types of sheet materials were tested and calculated. The results are shown in Figure 5 and Table 3 and Table 4.

According to Figure 5a, compared with the pulp-only sheet material, the contact angle of both CSAgNP-treated and chitosan-coated paper samples increased from 8.75° to 88.25° (the minimum at BP/Ch 0.5) to 89.53° (the maximum at BP/CSAgNPs 1). When compared with the BP water contact angle, a decrease in the range of 9.38° to 10.66° was observed, and the wettability increased respectively (Figure 5b). The hydrophobicity of the treated and coated papers was slightly deteriorated, but the effect was not higher than 10.7% and was very close to 90°, and thus they were considered hydrophobic. In addition to the hydrophilic or hydrophobic nature of the material tested, its granularity has an influence on the contact angle and wettability by reducing the exact values. The opposite trend was visible for the water absorbency Cobb_60_ values of the CSAgNPs treated samples, as presented in Table 3. This discrepancy in results is due to the significant difference in the scale of the two methods used. The first one depends on the micro-characteristics of the paper structure, and it uses deionized water, while for the second one, the macro-characteristics of the paper structure has a leading role, and the water used for the analysis is distilled, which creates a difference in surface tension between the water and the paper being tested. The difference in surface tension between deionized and distilled water can result in different wetting behaviors. Deionized water may have a slightly lower surface tension, leading to better penetration and spreading over the paper’s microstructure. By contrast, distilled water might have a slightly higher surface tension, affecting the macroscopic wetting pattern and possibly leading to different results when analyzing the paper’s macro-characteristics. Additionally, the calendaring effect as a result of paper drying poses the formation of a compacted paper surface structure with highly blocked hydroxyl groups as a result of the increased CSAgNPs consumption.

The effect of chitosan coatings on hydrophobicity was determined using two methods with three parameters—the water contact angle (Figure 5a), wettability (Figure 5b), and surface water absorption capacity (Cobb_60_, g/m^2^) (Table 3). 

Regarding the water barrier properties of papers coated with chitosan, a slight decrease in hydrophobicity was observed. The decrease in hydrophobicity remained minimal, and considering the unique sorption properties of chitosan and its ability to connect equally with either hydrophobic or hydrophilic substances, the obtained results should not be perceived as negative. Furthermore, when coating, the drying temperature was relatively low (50 °C) and the probability of the presence of invisible coating pores could increase, as some previous investigations have confirmed that chitosan fills the surface pores, and only some of it can adhere chemically to the surface [58]. The presence of chitosan on the surface of the paper samples might have led to a more uniform distribution of polar groups (hydroxyl and amino groups, which can form hydrogen bonds with water molecules), which can increase the wettability without significantly affecting the WCA. The distribution of surface energy might have become more favorable for wetting. WCA does not capture the entire picture of wettability behavior. Chitosan’s ability to interact with water through its hydrophilic groups and the potential changes in surface roughness and energy distribution likely contribute to the observed increase in wettability, even when the WCA remains relatively unchanged. Taking into account the SEM images and both the water contact angle and Cobb_60_, as presented above, we can conclude that 1 g/m^2^ chitosan coating is optimal for the complex hydrophobicity examined. When applying smaller or larger coating layer invisible pores, cracks and bombs could violate the uniformity of the sample’s surface structure.

According to the data from the three water resistance indicators (water contact angle, wettability, and Cobb_60_) and the SEM images, it could be expected that when applying a chitosan coating layer of more than 5 g/m^2^ over bleached kraft celluloses, it is not necessary to use all the wet-end chemical additives in the base paper but only those responsible for optimal functionality (as sizing agents) and excellent processability (such as retention and dewatering additives) since the hydrophobicity of the chitosan coated samples was very close to the BP (control). This would enhance chemical savings as well as cost efficiency and environmentally friendly paper production.

The OP sample showed a much higher wettability with a significantly lower contact angle (8.76°) and very consistent measurements compared with the BP control, which had a high contact angle (98.91°) and greater variability. Both the BP/CSAgNP and BP/Ch samples had reduced contact angles (around 89° for BP/CSAgNPs and 88.25° to 89.39° for BP/Ch) compared with the BP control, indicating decreased hydrophobicity. Their standard deviations were lower than those of the BP control but still showed noticeable variability. Within the BP/CSAgNPs and BP/Ch groups, the contact angles and standard deviations were very close, indicating consistent results within each group.

The OP sample had a small standard deviation, indicating consistent wettability measurements despite its high wettability (3.276°/s). The BP control sample had an extremely low standard deviation, showing highly consistent measurements with minimal variability despite its low wettability (0.107°/s). On the other hand, the BP/CSAgNP samples exhibited standard deviations that were slightly higher but still low compared with those of the BP control, suggesting some increased variability, but they remained consistent within the group. A noticeable increase in standard deviations compared with both the BP control and BP/CSAgNPs samples was shown in BP/CH samples, indicating greater variability in wettability measurements. The increasing standard deviation in the BP/Ch group correlated with a higher wettability, suggesting that modifications with Ch introduced more measurement variability compared with CSAgNP modification.

The Cobb_60_ values for the three batches of CSAgNPs treated papers were very close to each other, with values ranging from 18.54 g/m^2^ to 18.74 g/m^2^. The standard deviations were also low (±0.7, ±0.5, and ±0.5 respectively), indicating consistency in the treatment process. This consistency suggests that the CSAgNPs treatment produces reliable and reproducible effects on the paper’s water absorbency, with only minor variations between different batches.

Coating the base paper with chitosan slightly increased its water absorbency, but the effect was relatively modest. The increase in water absorbency did not linearly correlate with the concentration of chitosan, as seen with similar values for BP/Ch 0.5 and BP/Ch 2. The higher standard deviation in only pulp indicated greater variability, while the lower and similar standard deviations in the base paper and chitosan-coated papers suggested more consistent water absorbency characteristics. Low variability as the base paper indicated that the chitosan coating process was consistent and did not significantly alter the uniformity of the paper’s absorbency properties.

**Figure 6:**
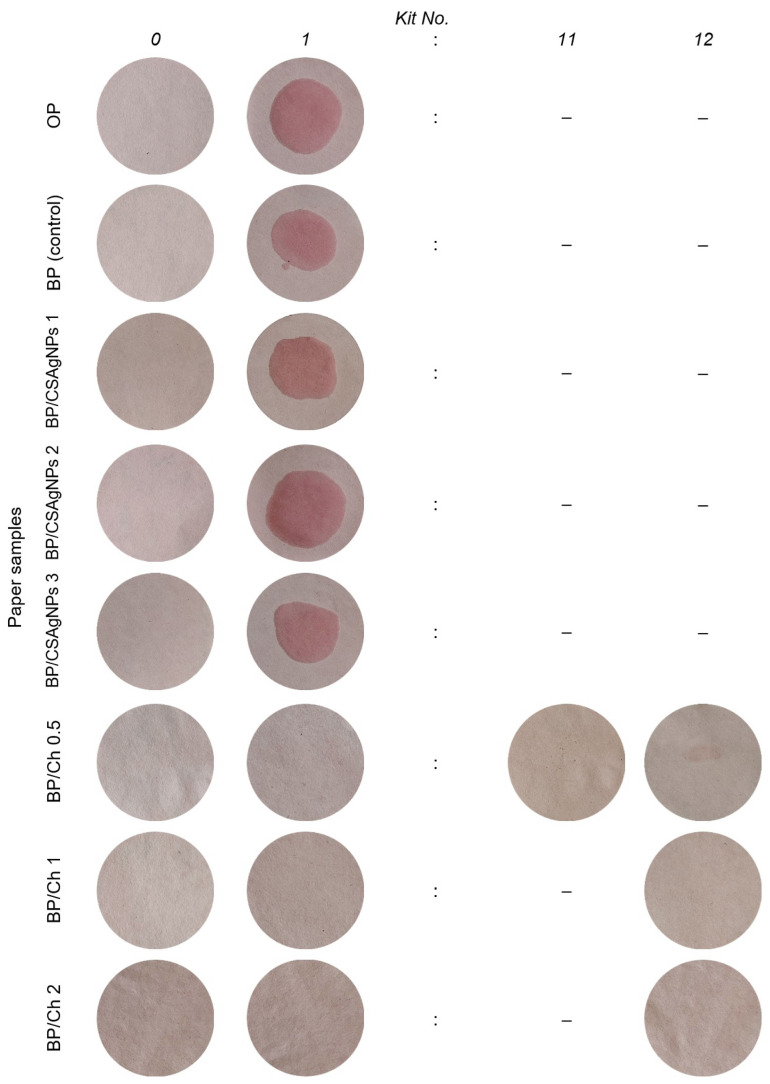
Grease resistance test (Kit Test) of the cellulose, base papers, CSAgNP-treated, and chitosan-coated paper samples.

The oil resistance of packaging papers, especially for those intended for contact with food, is essential for the consumer qualities of the final products, as it is of fundamental importance for the packaging industries. Figure 6 presents the results of the grease resistance test (Kit test) of the cellulose, base papers, and CSAgNP-treated and chitosan-coated paper samples. The Kit test is a classic standard method known by the manufacturers, and it is widely used in the papermaking practice due to the simplicity of its application and excellent accuracy regarding the oil resistance of the packaging materials.

The analysis of the results indicated a lack of grease resistance of the paper samples treated with CSAgNPs (wetting is observed even with the first Kit solution No. 1), as expected, meaning that the oil is absorbed by the paper. A major effect of CSAgNP treatment on packaging papers was expected to be found in the analysis of the antibacterial activity of the obtained papers (the results are given below in Section 3.3).

The chitosan coatings produced in the current experiment were highly grease-resistant. When increasing the chitosan coating layer grammage from 0.5 to 2 g/m^2^, the Kit value increased from Kit 1 (BP) to Kit 11 (BP/Ch 0.5), where slight wetting was observed, and Kit 12 (BP/Ch 1 and BP/Ch 2), which was the most aggressive solution failed to cause absorption. These results confirm the effectiveness of chitosan coatings in increasing the oil resistance of packaging papers, which has also been reported by other researchers and their investigations with chitosan coated packaging papers, reaching grease resistance in Kit 7 [59].

### 3.3. Antimicrobial Activity

Generally speaking, the inhibition efficiency of silver and its associated ions is due to the strong bonding with disulfide (S-S) and sulfhydryl (-SH) groups found in the microbial cell wall proteins. Through this binding, normal metabolic processes are disrupted, resulting in cell death. Thus, the antimicrobial metals silver (Ag), copper (Cu), and zinc (Zn) have found a higher scientific and research interest and antibacterial application [60,61]. 

The chitosan antibacterial activity mechanisms are described in rupturing the bacterial cell wall by the free amino groups, inhibiting microorganisms’ growth by chelating for metal ions, and interfering with the synthesis of mRNA and proteins by combining with the DNA penetrating microorganisms’ cytoplasm [62]. 

Both, CSAgNPs and chitosan, substances are investigated in the current experiment and the presented comparative analysis for nine microorganisms is shown in Table 5 and Table 6.

The inhibition efficiency results showed that 3 mL/20 cm^2^ CSAgNPs treated paper samples (BP/CSAgNPs 3) inhibited the growth of the Gram-positive bacterium *S. aureus* with 81.6% and the Gram-negative bacterium *E. coli* with 75.8%. The impact is weaker compared to Gram-negative *S. abony* (45.6%) bacterium and the yeast *S. cerevisiae* (51.2%). The antimicrobial activity of this type of paper against Gram-positive bacterium *B. cereus* (40.4%), *P. aeruginosa* (38.8%), yeast *C. albicans* (37.1%), fungal strain *A. brasiliensis* (35.5%) and *F. moniliforme* (36.9%) is around and below 40%. Treatment of the paper with 1 and 2 mL/20 cm^2^ of CSAgNPs (BP/CSAgNPs 1 and BP/CSAgNPs 2) is less effective, 27.13 and 39.83%, respectively.

From the results analysis of the CSAgNPs treatment increasing from BP/CSAgNPs 1 to BP/CSAgNPs 3, it was found that the treatment is most effective for the Gram-positive bacteria (with about 106%), followed by the Gram-negative with an efficiency of about 77% and 74.8% for the fungal strains and yeast from 70%. In the between-group analysis, the order of the group effectiveness is confirmed, except for the yeast and fungal strain, which exchange their positions.

By the surface chitosan film formation, antibacterial and fungicidal efficiency are obtained by the controlled anionic pulp charge, as a result of chitosan charge density and the amino groups in its molecular structure [63]. In this experiment, the inhibition efficiency of the applied chitosan coating of 2 g/m^2^ (BP/Ch 2) is the most effective (average 79%) against the tested bacteria, yeasts, and fungal strains. At 1 and 0.5 g/m^2^ chitosan coating (BP/Ch 1 and BP/Ch 0.5), the efficiency is 72.38% and 54.67%, respectively. 

Chitosan-coated paper samples with a 2 g/m^2^ coating layer inhibit the Gram-positive bacteria *S. aureus* and *B. cereus* and the Gram-negative bacterium *E. coli*, the yeasts *C. albicans* and *S. cerevisiae*, and the fungal strains *A. brasiliensis* and *F. moniliforme* by 80–90%. The impact of this coating layer is weaker against the Gram-negative bacteria *P. aeruginosa* (56.3%) and *S. ebony* (48.6%).

The 1 g/m^2^ chitosan layer is found to be about 80% effective against the Gram-positive bacteria *S. aureus*, *B. cereus*, the Gram-negative bacterium *E. coli*, the yeasts *C. albicans*, *S. cerevisiae* and the fungal strain *A. brasiliensis* and *F. moniliforme*. The inhibition efficiency of the 1 g/m^2^ chitosan layer is lower against the Gram-negative bacteria *P. aeruginosa* (54.8%) and *S. ebony* (47.4%).

Lower inhibition efficiency is observed with the lowest applied coating layer of 0.5 g/m^2^. These results could be summarized in the statement that as a lower chitosan coating layer is applied, the inhibition efficiency is expected to be lower. 

Between-group analysis on the average inhibition effectiveness shows that the obtained chitosan coating is most effective for the yeast, followed by the fungal strain, Gram-positive and Gram-negative bacteria, while with increasing the chitosan coating layer from 0.5 g/m^2^ to 2 g/m^2^ the effectiveness (expressed in %) is having the highest values for the Gram-negative bacteria with 86.42%, followed by the Gram-positive bacteria with 62.36% and 23.65% and 20.55% for the yeast and fungal strain, respectively.

## 4. Conclusions

This study compared the CSAgNPs treatment and chitosan coating on the barrier properties of laboratory-obtained packaging paper. In terms of hydrophobicity (Cobb_60_), all examined paper samples could be ranked as follows: BP/CSAgNPs 1 > BP/CSAgNPs 2 > BP/CSAgNPs 3 > BP (control) > BP/Ch 1 > BP/Ch 0.5 > BP/Ch 2 > OP. The analysis of the results indicated a lack of grease resistance of the paper samples treated with CSAgNPs. The chitosan coatings produced in the current experiment are highly grease-resistant. When increasing the chitosan coating layer grammage from 0.5 to 2 g/m^2^, the Kit value increased from Kit 1 (BP) to Kit 11 (BP/Ch 0.5), where slight wetting is observed, and Kit 12 (BP/Ch 1 and BP/Ch 2), which was the most aggressive solution, failed to cause absorption. Additionally, paper treated with 3 mL/20 cm^2^ of CSAgNPs had the highest antibacterial protection (81.6%) against the Gram-positive bacterium *S. aureus*, followed by Gram-negative *E. coli* (75.8%). For the rest of the studied microorganisms, the average efficiency of the treated paper was 40.79%. The treatment of the paper with 1 and 2 mL/20 cm^2^ of CSAgNPs was less effective, at 27.13 and 39.83%, respectively. The antibacterial protection of 2 g/m^2^ chitosan coated paper samples was the most effective (average 79%) against the tested bacterial, yeast, and fungal strains. For the 1 and 0.5 g/m^2^ chitosan coatings, the efficiencies were 72.38% and 54.67%, respectively. Gram-positive bacteria, yeasts, and fungal strains were more sensitive to chitosan supplementation.

## Figures and Tables

**Table 1:** Composition of investigated paper samples.

Paper Samples	Description	CSAgNP Treatment (mL/20 cm^2^)	Chitosan Coating (g/m^2^)
OP	Mixture of only pulp	-	-
BP (control)	Base paper	-	-
BP/CSAgNPs 1	Base paper treated with CSAgNPs	1	-
BP/CSAgNPs 2		2	-
BP/CSAgNPs 3		3	-
BP/Ch 0.5	Base paper coated with chitosan	-	0.5
BP/Ch 1		-	1
BP/Ch 2		-	2

**Table 2:** Grammage, thickness, and smoothness of all paper samples.

Paper Samples	Grammage (g/m^2^)	Thickness (mm)	Smoothness (Bekk, Top Side) (s)
OP	50.89 ± 0.3	0.081 ± 0.02	10.98 ± 1.13
BP (control)	50.96 ± 0.2	0.081 ± 0.01	10.96 ± 1.11
BP/CSAgNPs 1	50.48 ± 0.5	0.080 ± 0.03	14.89 ± 1.29
BP/CSAgNPs 2	49.68 ± 0.5	0.071 ± 0.03	14.92 ± 1.25
BP/CSAgNPs 3	49.95 ± 0.7	0.070 ± 0.03	14.03 ± 1.18
BP/Ch 0.5	50.48 ± 0.5	0.080 ± 0.02	12.27 ± 1.10
BP/Ch 1	49.68 ± 0.4	0.079 ± 0.02	14.56 ± 1.26
BP/Ch 2	49.95 ± 0.4	0.083 ± 0.03	16.42 ± 1.32

**Table 3:** Water absorbency Cobb_60_, g/m^2^ of only pulp, base paper, and CSAgNP-treated papers.

**Cobb_60_ (g/m^2^)**	**OP**	**BP**	**BP/CSAgNPs 1**	**BP/CSAgNPs 2**	**BP/CSAgNPs 3**
	68.81 ± 2.2	20.92 ± 1.2	18.54 ± 0.7	18.63 ± 0.5	18.74 ± 0.5

**Table 4:** Water absorbency Cobb_60_, g/m^2^ of only pulp, base paper, and chitosan-coated papers.

**Cobb_60_ (g/m^2^)**	**OP**	**BP**	**BP/Ch 0.5**	**BP/Ch 1**	**BP/Ch 2**
	68.81 ± 2.2	20.92 ± 1.2	21.65 ± 1.1	22.16 ± 1.2	21.66 ± 1.1

**Table 5:** Inhibition efficiency of the CSAgNP-treated paper samples against Gram-positive and Gram-negative bacteria, yeasts, and fungal strains.

Sample	Tested Microorganisms		Inhibition Efficiency of CSAgNPs Treated (%)		
			BP/CSAgNPs 1	BP/CSAgNPs 2	BP/CSAgNPs 3
A	Gram-positive bacteria	*Staphylococcus aureus* ATCC 6538	47.5	67.3	81.6
B		*Bacillus cereus* ATCC 10876	11.7	31.5	40.4
C	Gram-negative bacteria	*Escherichia coli* ATCC 8739	46.8	66.0	75.8
D		*Pseudomonas* *aeruginosa* ATCC 9027	16.3	20.4	38.8
E		*Salmonella abony* NTCC 6017	27.7	35.7	45.6
F	Yeast	*Candida albicans* ATCC 10231	18.6	34.4	37.1
G		*Saccharomyces cerevisiae* ATCC 2601	33.4	41.1	51.2
H	Fungal strain	*Aspergillus brasiliensis* ATCC 16404	15.1	28.3	35.5
I		*Fusarium moniliforme*	27.1	33.8	36.9

**Table 6:** Inhibition efficiency of the chitosan-coated paper samples against Gram-positive and Gram-negative bacteria, yeasts, and fungal strains.

Sample	Tested Microorganisms		Inhibition Efficiency of Chitosan Coating (%)		
			BP/Ch 0.5	BP/Ch 1	BP/Ch 2
A	Gram-positive bacteria	*Staphylococcus aureus* ATCC 6538	76.4	78.5	86.5
B		*Bacillus cereus* АТСС 10876	31.2	79.5	88.2
C	Gram-negative bacteria	*Escherichia coli* ATCC 8739	51.8	73.7	89.5
D		*Pseudomonasaeruginosa* ATCC 9027	32.3	54.8	56.3
E		*Salmonella abony* NTCC 6017	20.2	47.4	48.6
F	Yeast	*Candida albicans* ATCC 10231	62.9	81.7	82.6
G		*Saccharomyces cerevisiae* ATCC 2601	76.6	82.8	89.9
H	Fungal strain	*Aspergillus brasiliensis* ATCC 16404	65.9	70.1	82.4
I		*Fusarium moniliforme*	74.7	82.9	87.1

## Data Availability

Data are contained within the article.

## References

[1] Trinh B.M., Chang B.P., Mekonnen T.H. (2023). The Barrier Properties of Sustainable Multiphase and Multicomponent Packaging Materials: A Review. Prog. Mater. Sci..

[2] Perera K.Y., Jaiswal A.K., Jaiswal S. (2023). Biopolymer-Based Sustainable Food Packaging Materials: Challenges, Solutions, and Applications. Foods.

[3] De Matteis V., Cascione M., Costa D., Martano S., Manno D., Cannavale A., Mazzotta S., Paladini F., Martino M., Rinaldi R. (2023). Aloe Vera Silver Nanoparticles Addition in Chitosan Films: Improvement of Physicochemical Properties for Eco-Friendly Food Packaging Material. J. Mater. Res. Technol..

[4] Kumar S., Basumatary I.B., Sudhani H.P.K., Bajpai V.K., Chen L., Shukla S., Mukherjee A. (2021). Plant Extract Mediated Silver Nanoparticles and Their Applications as Antimicrobials and in Sustainable Food Packaging: A State-of-the-Art Review. Trends Food Sci. Technol..

[5] Riseh R.S., Vatankhah M., Hassanisaadi M., Kennedy J.F. (2023). Chitosan-Based Nanocomposites as Coatings and Packaging Materials for the Postharvest Improvement of Agricultural Product: A Review. Carbohydr. Polym..

[6] Alimi B.A., Pathania S., Wilson J., Duffy B., Frias J.M.C. (2023). Extraction, Quantification, Characterization, and Application in Food Packaging of Chitin and Chitosan from Mushrooms: A Review. Int. J. Biol. Macromol..

[7] Kumar-Krishnan S., Prokhorov E., Hernández-Iturriaga M., Mota-Morales J.D., Vázquez-Lepe M., Kovalenko Y., Sanchez I.C., Luna-Bárcenas G. (2015). Chitosan/silver nanocomposites: Synergistic antibacterial action of silver nanoparticles and silver ions. Eur. Polym. J..

[8] Morais L.D.O., Macedo E.V., Granjeiro J.M., Delgado I.F. (2020). Critical Evaluation of Migration Studies of Silver Nanoparticles Present in Food Packaging: A Systematic Review. Crit. Rev. Food Sci. Nutr..

[9] Ediyilyam S., George B., Shankar S.S., Dennis T.T., Wacławek S., Černík M., Padil V.V.T. (2021). Chitosan/Gelatin/Silver Nanoparticles Composites Films for Biodegradable Food Packaging Applications. Polymers.

[10] Wang W., Yu Z., Alsammarraie F.K., Kong F., Lin M., Mustapha A. (2020). Properties and Antimicrobial Activity of Polyvinyl Alcohol-Modified Bacterial Nanocellulose Packaging Films Incorporated with Silver Nanoparticles. Food Hydrocoll..

[11] Wang L., Periyasami G., Aldalbahi A., Fogliano V. (2021). The Antimicrobial Activity of Silver Nanoparticles Biocomposite Films Depends on the Silver Ions Release Behaviour. Food Chem..

[12] Brito S.d.C., Bresolin J.D., Sivieri K., Ferreira M.D. (2020). Low-density polyethylene films incorporated with silver nanoparticles to promote antimicrobial efficiency in food packaging. Food Sci. Technol. Int..

[13] Lazić V., Vivod V., Peršin Z., Stoiljković M., Ratnayake I.S., Ahrenkiel P.S., Nedeljković J.M., Kokol V. (2020). Dextran-Coated Silver Nanoparticles for Improved Barrier and Controlled Antimicrobial Properties of Nanocellulose Films Used in Food Packaging. Food Packag. Shelf Life.

[14] Istiqola A., Syafiuddin A. (2020). A Review of Silver Nanoparticles in Food Packaging Technologies: Regulation, Methods, Properties, Migration, and Future Challenges. J. Chin. Chem. Soc..

[15] Liu J., Ma Z., Liu Y., Zheng X., Pei Y., Tang K. (2022). Soluble Soybean Polysaccharide Films Containing In-Situ Generated Silver Nanoparticles for Antibacterial Food Packaging Applications. Food Packag. Shelf Life.

[16] Adel A.M., Al-Shemy M.T., Diab M.A., El-Sakhawy M., Toro R.G., Montanari R., De Caro T., Caschera D. (2021). Fabrication of Packaging Paper Sheets Decorated with Alginate/Oxidized Nanocellulose-silver Nanoparticles Bio-Nanocomposite. Int. J. Biol. Macromol..

[17] Yang D., Liu Q., Gao Y., Wan S., Meng F., Weng W., Zhang Y. (2023). Characterization of Silver Nanoparticles Loaded Chitosan/Polyvinyl Alcohol Antibacterial Films for Food Packaging. Food Hydrocoll..

[18] Liao M., Pan Y., Fu X., Wu S., Gan S., Wu Z., Zhao H., Zheng W., Cao Y., Zhou W. (2023). Electrospun Polylactic Acid Nanofiber Film Modified by Silver Oxide Deposited on Hemp Fibers for Antibacterial Fruit Packaging. Int. J. Biol. Macromol..

[19] Mulla M.Z., Rahman M.R.T., Marcos B., Tiwari B., Pathania S. (2021). Poly Lactic Acid (PLA) Nanocomposites: Effect of Inorganic Nanoparticles Reinforcement on Its Performance and Food Packaging Applications. Molecules.

[20] Samrot A.V., Samanvitha S.K., Shobana N., Renitta E.R., Senthilkumar P., Kumar S.S., Abirami S., Dhiva S., Bavanilatha M., Prakash P. (2021). The Synthesis, Characterization and Applications of Polyhydroxyalkanoates (PHAs) and PHA-Based Nanoparticles. Polymers.

[21] He Y., Li H., Fei X., Peng L. (2021). Carboxymethyl Cellulose/Cellulose Nanocrystals Immobilized Silver Nanoparticles as an Effective Coating to Improve Barrier and Antibacterial Properties of Paper for Food Packaging Applications. Carbohydr. Polym..

[22] Zhang J., Cao C., Wang Y., Xie L., Li W., Li B., Guo R., Yan H. (2021). Magnesium Oxide/Silver Nanoparticles Reinforced Poly(Butylene Succinate-Co-Terephthalate) Biofilms for Food Packaging Applications. Food Packag. Shelf Life.

[23] Kraśniewska K., Galus S., Gniewosz M. (2020). Biopolymers-Based Materials Containing Silver Nanoparticles as Active Packaging for Food Applications—A Review. Int. J. Mol. Sci..

[24] Chen K., Wang F., Liu S., Wu X., Xu L., Zhang D. (2020). In Situ Reduction of Silver Nanoparticles by Sodium Alginate to Obtain Silver-Loaded Composite Wound Dressing with Enhanced Mechanical and Antimicrobial Property. Int. J. Biol. Macromol..

[25] Zhang W., Hadidi M., Karaca A.C., Hedayati S., Tarahi M., Assadpour E., Jafari S.M. (2023). Chitosan-Grafted Phenolic Acids as an Efficient Biopolymer for Food Packaging Films/Coatings. Carbohydr. Polym..

[26] Purohit S.D., Priyadarshi R., Bhaskar R., Han S.S. (2023). Chitosan-Based Multifunctional Films Reinforced with Cerium Oxide Nanoparticles for Food Packaging Applications. Food Hydrocoll..

[27] Barik M., BhagyaRaj G.V.S., Dash K.K., Shams R. (2024). A Thorough Evaluation of Chitosan-Based Packaging Film and Coating for Food Product Shelf-Life Extension. J. Agric. Food Res..

[28] Vrabič-Brodnjak U., Yavorov N., Lasheva V., Todorova D. (2023). Chitosan-Coated Packaging Papers—Strength and Thermal Stability. Coatings.

[29] Harikrishnan M.P., Thampi A., Lal A.M.N., Warrier A.S., Basil M., Kothakota A. (2023). Effect of Chitosan-Based Bio Coating on Mechanical, Structural and Physical Characteristics of Microfiber Based Paper Packaging: An Alternative to Wood Pulp/Plastic Packaging. Int. J. Biol. Macromol..

[30] Liu F., Zhang X., Xiao X., Duan Q., Bai H., Cao Y., Zhang Y., Alee M., Yu L. (2023). Improved Hydrophobicity, Antibacterial and Mechanical Properties of Polyvinyl Alcohol/Quaternary Chitosan Composite Films for Antibacterial Packaging. Carbohydr. Polym..

[31] Khanzada B., Mirza B., Ullah A. (2023). Chitosan Based Bio-Nanocomposites Packaging Films with Unique Mechanical and Barrier Properties. Food Packag. Shelf Life.

[32] Bhardwaj S., Kaur P., Bhardwaj N.K., Negi Y.S. (2023). Surface Application of Different Concentrations of Chitosan on Recycled Paper and Its Impact on Packaging Properties. J. Coat. Technol. Res..

[33] Nazarnezhad N., Avrand M., Resalati H. (2023). The Effect of Chitosan Coating on the Strength and Barrier Properties of Liner Paper. Iran. J. Wood Pap. Sci. Res..

[34] Cheng Z., Li J., Su M., Xiao N., Zhong L., Zhang X., Chen S., Chen Q., Liang W., Liu M. (2024). High-Barrier Oxidized Cellulose Nanofibril/Chitosan Coating for Functional Food Packaging Materials. ACS Appl. Polym. Mater..

[35] Inthamat P., Karbowiak T., Tongdeesoontorn W., Siripatrawan U. (2024). Biodegradable Active Coating from Chitosan/Astaxanthin Crosslinked with Genipin to Improve Water Resistance, Moisture and Oxygen Barrier and Mechanical Properties of Kraft Paper. Int. J. Biol. Macromol..

[36] Gürler N. (2023). Development of Chitosan/Gelatin/Starch Composite Edible Films Incorporated with Pineapple Peel Extract and Aloe Vera Gel: Mechanical, Physical, Antibacterial, Antioxidant, and Sensorial Analysis. Polym. Eng. Sci..

[37] Shan P., Wang K., Yu F., Yi L., Sun L., Li H. (2023). Gelatin/Sodium Alginate Multilayer Composite Film Crosslinked with Green Tea Extract for Active Food Packaging Application. Colloids Surf. A.

[38] Rohadi T.N.T., Ridzuan M.J.M., Majid M.S.A., Sulaiman M.H. (2023). Biodegradability of Bioplastic Film Using Different Regions of Pennisetum Purpureum Incorporated with Gelatine and Chitosan. Int. J. Environ. Sci. Technol..

[39] Wawrzyńczak A., Chudzińska J., Feliczak-Guzik A. (2024). Metal and Metal Oxides Nanoparticles as Nanofillers for Biodegradable Polymers. ChemPhysChem.

[40] Saleem S., Khan S.T. (2023). Engineered Nanomaterials for Food Preservation and Packaging: Focus on Antimicrobial and Antibiofilm Activities. Quality Control in Fruit and Vegetable Processing.

[41] Sharma A., Sharma A., Baurai M., Loria N., Dubey N., Tokala V.Y. (2023). Implications of Nanotechnology in Food Packaging. Nanotechnology Horizons in Food Process Engineering.

[42] Advaita, Malik K., Bhagabati V.P., Sharma K. (2023). Nanostructured Film and Coating Materials. Biopolymer-Based Films and Coatings.

[43] Cherian R.M., Antony T., Zakuwan S.Z., Jose C., Kargarzadeh H., Thomas S. (2022). Developments in Chitosan-Based Nanocomposites for Food Packaging Applications. Nanomaterials from Renewable Resources for Emerging Applications.

[44] Sharma H., Ahuja A., Sharma B., Kulshreshtha A., Kadam A., Dutt D. (2024). Vapor Phase Antimicrobial Active Packaging Application of Chitosan Capsules Containing Clove Essential Oil for the Preservation of Dry Cakes. Food Bioprocess Technol..

[45] (1979). Pulps—Laboratory Beating, Part 1: Valley Beater Method.

[46] (2001). Pulps—Determination of Drainability—Part 1: Schopper-Riegler methodTechnical Corrigendum 1.

[47] (2004). Pulps—Preparation of Laboratory Sheets for Physical Testing—Part 2: Rapid-Köthen Method.

[48] (2021). Paper, Board and Pulps—Fibre Furnish Analysis.

[49] (2019). Paper and Board—Determination of Grammage.

[50] (2022). Paper and Board—Determination of Thickness, Density and Specific Volume.

[51] (2002). Paper and Board—Determination of Smoothness (Bekk Method)—Technical Corrigendum 1.

[52] (2009). Standard Test Method for Surface Wettability of Paper (Angle-of-Contact Method).

[53] (2023). Paper and Board—Determination of Water Absorptiveness—Cobb Method.

[54] Salmén L., Larsson P.A. (2018). On the Origin of Sorption Hysteresis in Cellulosic Materials. Carbohydr. Polym..

[55] Kjellgren H., Gällstedt M., Engström G., Järnström L. (2006). Barrier and Surface Properties of Chitosan-Coated Greaseproof Paper. Carbohydr. Polym..

[56] Gal M.R., Rahmaninia M., Hubbe M.A. (2023). A Comprehensive Review of Chitosan Applications in Paper Science and Technologies. Carbohydr. Polym..

[57] Song H., Choi I., Lee J.-S., Chang Y., Yoon C.S., Han J. (2022). Whey Protein Isolate Coating Material for High Oxygen Barrier Properties: A Scale-up Study from Laboratory to Industrial Scale and Its Application to Food Packaging. Food Packag. Shelf Life.

[58] Zakaria S., Chin H.C., Wan Ahmad W.H., Kaco H., Soon W.C., Chi H.C. (2015). Mechanical and Antibacterial Properties of Paper Coated with Chitosan. Sains Malays..

[59] Bordenave N., Grelier S., Coma V. (2010). Hydrophobization and Antimicrobial Activity of Chitosan and Paper-Based Packaging Material. Biomacromolecules.

[60] Dakal T.C., Kumar A., Majumdar R.S., Yadav V. (2016). Mechanistic Basis of Antimicrobial Actions of Silver Nanoparticles. Front. Microbiol..

[61] More P.R., Pandit S., Filippis A.D., Franci G., Mijakovic I., Galdiero M. (2023). Silver Nanoparticles: Bactericidal and Mechanistic Approach against Drug Resistant Pathogens. Microorganisms.

[62] Wang W., Meng Q., Li Q., Liu J., Zhou M., Jin Z., Zhao K. (2020). Chitosan Derivatives and Their Application in Biomedicine. Int. J. Mol. Sci..

[63] Andersson C. (2008). New Ways to Enhance the Functionality of Paperboard by Surface Treatment—A Review. Packag. Technol. Sci..

